# Use of UK national health databases for detecting intra-cranial aneurysm rupture in the Risk of Aneurysm Rupture (ROAR) study

**DOI:** 10.1371/journal.pdig.0001388

**Published:** 2026-05-18

**Authors:** Samuel Hall, Jacqueline Birks, Frederick Ewbank, Benjamin Gaastra, Girjia Agarwal, Harsh Bhatt, Guendalina Bonifacio, Annabel Dainton, Alexander Dando, Mohamed Elmarawany, Cathal Hannan, Lauren Harris, Matthew Harris, Josephine Jung, Nida Kalyal, James JM Loan, David Lowes, Haritha Maripi, Mustafa Motiwala, Matthew Myers, Frazer O’Brien, Ikenna Ogbu, William Parton, David Rowland, Fozia Saeed, Eleanor Taylor, Sara Venturini, Diederik Bulters, on behalf of the ROAR Collaborative

**Affiliations:** 1 Department of Neurosurgery, University Hospitals Southampton NHS Foundation Trust, Southampton, Hampshire, United Kingdom; 2 Centre for Statistics in Medicine, Medical Sciences Division, University of Oxford, Oxford, Oxfordshire, United Kingdom; 3 Department of Radiology, Imperial College Healthcare NHS Trust, London, United Kingdom; 4 Neurosurgical Department, University Hospital Wales, Cardiff, United Kingdom; 5 Department of Neurology, University Hospitals Southampton NHS Foundation Trust, Southampton, Hampshire, United Kingdom; 6 Department of Neurosurgery, Northern Care Alliance NHS Foundation Trust, Salford, United Kingdom; 7 Department of ENT surgery, University Hospitals Dorset, Poole, Dorset, United Kingdom; 8 Department of Neurosurgery, North Bristol NHS Trust, Bristol, Avon, United Kingdom; 9 Department of Neurosurgery, Walton Centre NHS Foundation Trust, Liverpool, Merseyside, United Kingdom; 10 Department of Neurosurgery, Barking Havering and Redbridge University Hospitals NHS Trust, London, United Kingdom; 11 Department of Neurology, Hampshire Hospitals NHS Foundation Trust, Winchester, Hampshire, United Kingdom; 12 Department of Neurosurgery, King’s College Hospital NHS Foundation Trust, London, United Kingdom; 13 Institute of Psychiatry, Psychology & Neuroscience, King’s College London, London, United Kingdom; 14 Department of Neurosurgery, University Hospitals Sussex NHS Foundation Trust, Brighton, West Sussex, United Kingdom; 15 Department of Clinical Neurosciences, NHS Lothian, Edinburgh, Lothian, United Kingdom; 16 South West Neurosurgery Centre, University Hospitals Plymouth NHS Trust, Plymouth, Devon, United Kingdom; 17 Department of Neurosurgery, Sheffield Teaching Hospitals NHS Foundation Trust, Sheffield, Yorkshire, United Kingdom; 18 Department of Neurosurgery, Lancashire Teaching Hospitals NHS Foundation Trust, Preston, Lancashire, United Kingdom; 19 Department of Neurosurgery, University Hospitals of North Midlands NHS Trust, Stoke-on-Trent, Staffordshire, United Kingdom; 20 Department of Emergency Medicine, Hampshire Hospitals NHS Foundation Trust, Winchester, Hampshire, United Kingdom; 21 Department of Neurosurgery, Barts Health NHS Trust, London, United Kingdom; 22 Department of Neurosurgery, The Leeds Teaching Hospitals NHS Trust, Leeds, Yorkshire, United Kingdom; 23 Department of Neurosurgery, Cambridge University Hospitals NHS Foundation Trust, Cambridge, Cambridgeshire, United Kingdom; Massachusetts Institute of Technology, UNITED STATES OF AMERICA

## Abstract

The objective of this study was to determine the sensitivity of national databases for identifying aneurysm rupture events in patients with unruptured intracranial aneurysms and determine their suitability for follow-up for patients in the Risk of Aneurysm Rupture (ROAR) Study. ROAR is a longitudinal cohort study that has recruited 20,000 patients with unruptured intracranial aneurysms with detailed baseline clinical and imaging data collected for each participant. These patients will be followed-up using UK national databases for hospital admissions (HES-APC) and national databases of deaths (CRD) to identify aneurysm rupture events for the purpose of rupture risk prediction. To assess the suitability of national databases for this, a cohort of patients with unruptured intracranial aneurysms was identified at a single neurosurgery centre from records between 2006–2020. Patients were linked to the national databases to identify instances containing intracranial haemorrhage diagnosis codes. All returned hospital admissions underwent case note and CT scan review to confirm the true diagnosis or cause of death. Of 1,544 patients, 74 were identified to have suffered a subsequent aneurysmal rupture. The national databases of hospital admissions and deaths identified 57 hospital admissions for aneurysm rupture. The national database of deaths identified an additional 16 out-of-hospital deaths due to aneurysm rupture of which 11 (68.8%) were confirmed on post-mortem. Local hospital records identified one additional inpatient admission for aneurysm rupture. Based on the observed proportions of admissions missing from the national databases and local hospital records, an estimated 1.03 admissions for rupture were predicted to be missed by both. The estimated sensitivity of a national database search strategy for identifying admissions for aneurysm rupture was 96.6%, and 98.3% if combined with local hospital records. National databases can detect rupture events in patients with unruptured intracranial aneurysms with high sensitivity and are ideally suited to long-term follow-up in large longitudinal cohort studies.

## Author summary

Patients with unruptured intracranial aneurysms rely on natural history data to estimate their future rupture risk and so guide their treatment decision making. In this study we aimed to measure the sensitivity of national digital databases in the UK for identifying aneurysm rupture events in patients with a previously known unruptured aneurysm. A cohort of 1,544 patients with an unruptured intracranial aneurysm were linked to national databases of hospital admissions and deaths for events that could possibly indicate an aneurysm rupture (subarachnoid haemorrhage (SAH), intracerebral (ICH), subdural haemorrhage (SDH), traumatic subarachnoid haemorrhage (tSAH), and sudden death). The results show a high sensitivity for these databases to identify rupture events. SAH codes alone would miss 6 cases found under ICH codes and 1 under SDH codes. These codes can therefore be used to screen for cases of aneurysm rupture that can then be confirmed with manual case note and imaging review to remove false positives and maintain specificity. The demonstrated high sensitivity of national digital databases of healthcare records supports their use as a screening tool for identifying potential aneurysm rupture events in the Risk of Aneurysm Rupture Study.

## Introduction

Unruptured intracranial aneurysms (UIA) are common with a prevalence of 3.2% [1] in the adult population. Although they provoke a lot of anxiety, their short-term rupture rates are relatively low at around 1% per annum [2]. Ideally the decision whether to treat a UIA or not would be based on long term rupture risk and be personalised to reflect individual patient and aneurysm features. However, very little long term natural history data is available with the largest long-term cohort comprising only 142 patients. [3] Instead, most of the available data comes from short-term studies whose risk estimates vary considerably. This variation is amplified when extrapolated to estimate long-term risks. Obtaining more accurate and more personalised risk estimates is difficult and costly due to low event rates necessitating large cohorts with long periods of follow-up.

The Risk of Aneurysm Rupture (ROAR) Study is a national, multicentre study to address the natural history of UIA. [4] The study has recruited more than 20,000 patients with individual patient level data to evaluate common, as well as uncommon but clinically important, covariates. To collect prospective outcomes over long periods of follow-up in such a large cohort, the ROAR Study will use electronic healthcare databases with nationwide coverage to identify rupture events. In England, NHS England (formerly NHS Digital) maintains Hospital Episode Statistics (HES) and the Office of National Statistics (ONS) maintains Civil Registrations of Death (CRD) databases which are both national, prospectively maintained records of all hospital admissions and deaths throughout England. [5] Each database entry contains the ICD-10 diagnosis codes relating to that admission or death, and are searchable using patient identifiable details.

The accuracy of the ROAR Study is dependent on the ability of these databases to identify rupture events in patients with known UIA. Many clinicians are sceptical about the accuracy of such databases for informing individual patient management. This is understandable when systematic reviews of databases internationally have shown variation in accuracy of aneurysmal subarachnoid haemorrhage (SAH) diagnostic codes between 46 and 100% [6–11]. However, Hospital Episode Statistics Admitted Patient Care (HES-APC) appears to perform at the upper end of this with two different studies demonstrating that the ICD-10 code I60 has a positive predictive value of 96% for aneurysmal subarachnoid haemorrhage [12,13]. The HES-APC database has also demonstrated high levels of accuracy in other neurosurgical fields such as pituitary surgery and overall neurosurgical mortality. [14,15]

Although these positive predictive values are good, they do not measure false negatives. The majority of patients in a longitudinal study of UIA won’t experience SAH, so even a small false omission rate can result in significant inaccuracy. Recognising this, it was decided in ROAR to use HES-APC and CRD as a screening tool to identify cases of possible SAH which will all undergo CT scan review (and where not performed case note or autopsy review) to confirm diagnosis.

The aim of this manuscript is to test this strategy in a single centre series, and describe the ability of HES-APC and CRD databases to detect rupture events in a cohort of patients with known UIA.

## Methods

### Patient population

Patients from one of the 27 centres participating in ROAR were used for this study. Patients with UIA were identified using a keyword search for the term “aneurysm” from Electronic Patient Records (EPR) at the University Hospital Southampton NHS Foundation Trust (UHS). The UHS EPR contains outpatient clinic letters, discharge summaries, operation notes, Multidisciplinary Team (MDT) discussions, and other patient correspondence. EPR records from 1/1/2006–31/12/2020 were retrospectively searched and, only department of neuroscience records were screened to confirm patient eligibility as described in the ROAR Study protocol [4]. Patients were ascribed a recruitment date based on the date of the document from which their UIA was identified. Patients’ resident in the Channel Islands were excluded due to inconsistent recording in NHS England. Research Ethics Committee (21/SC/0064) and Confidentiality Advisory Group approval (21CAG0033) were obtained to use patient data without consent for the ROAR Study.

### Data linkage

All eligible patients identified at UHS with UIA were linked by NHS England to the HES-APC and CRD databases to identify all possible rupture events treated in hospital or who died pre-hospital. Patient name, date of birth, NHS number, post code and gender were used for this linkage. Full details on NHS England’s patient linkage algorithms are available on their website [16]. HES-APC and CRD datasets for financial years 2005/06–2022/23 were linked.

HES-APC episodes provided were limited by NHS England to those that contained an ICD-10 diagnosis code for aneurysmal subarachnoid haemorrhage (I60), intra-cerebral haemorrhage (I61), subdural haemorrhage (I62) or traumatic subarachnoid haemorrhage (S06) in any of the 20 DIAG_01 > DIAG_20 diagnosis positions. The inclusion of intra-cranial haemorrhage codes in addition to I60 aimed to capture admissions for aneurysm rupture that were miscoded. It is also necessary to mitigate the inconsistency in ICD-10 where it is possible to have a pure ICH or SDH from a ruptured aneurysm which would be correctly coded as I61 or I62 with no way to identify if these were of aneurysmal origin. Although such instances of “pure ICH” and “pure SDH” are relatively rare, mixed ICH and SAH is relatively common. Although we might expect such patients to receive both an I60 and I61 code there is no clear guidance on this and application of this may not be consistent. Codes for cardiac arrest (I46), instantaneous death (R96) or unattended death (R98) were included to capture events of sudden death. Linkage to the CRD database returned all-cause mortality for the cohort.

### HES-APC

Only episodes starting (data element: EPISTART) on or after the date of inclusion of the aneurysm into the ROAR Study were subject to review for diagnosis confirmation. A continuous inpatient spell was defined as a series of episodes for the same patient with contiguous EPISTART and EPIEND dates. A continuous inpatient spell is thus considered a hospital admission spanning one or multiple hospital sites ending in discharge to the community, or death. Every continuous inpatient spell underwent manual stepwise review by the lead author (SH) to determine the single diagnosis responsible for the admission. A stepwise review comprised UHS EPR, radiology imaging, and then external hospital medical records. EPR confirmed episodes of index SAH, repatriations for SAH rehabilitation, or treatment of post-haemorrhagic hydrocephalus did not require further review. CT imaging with evidence of either basal cistern SAH, intra-parenchymal, or subdural haemorrhage, consistent with the UIA location was classed as UIA rupture. Rupture events found in EPR also underwent CT review. CT findings consistent with a diagnosis code other than I60 were classed as unruptured and not reviewed further. In those patients requiring a case note review, a sudden collapse with sustained coma (resulting in death before CT scan) was deemed SAH.

### CRD

Within the CRD database a patient’s cause of death is recorded using ICD-10 diagnosis codes in seven data elements named Cause Of Death (COD) 1–7. Diagnoses are assigned to COD 1 to COD7 sequentially from the Medical Certificate of Cause of Death 1a-2 position. COD_UNDERLYING data element recording the single pathology initiating any other cause of death as assigned per WHO International Statistical Classification of Disease and Related Health Problems. Patients whose place of death was in hospital (data element POD-ESTABLISHMENT_TYPE = 1,3,18,19,99) with cause of death, either COD1–7 or COD_UNDERLYING, containing a haemorrhage diagnosis code (I60, I61, I62, S06) or sudden death codes (I46, R96, R98) underwent review of UHS EPR, radiology imaging and medical records as per HES-APC above. Where imaging was not performed confirmation of whether a Post-Mortem (PM) had taken place was done with the hospital’s bereavement care services.

For patients in the UHS cohort who died out of hospital with any of the haemorrhage codes (I60, I61, I62, S06), inpatient notes and radiology were again checked for recent events, and the county coroner’s offices contacted to find out whether a post-mortem took place.

The UK Coroners and Justice Act 2009 dictates that any unexplained death and/or where a patient was not attended by a doctor in their final illness must be reported to the coroner and the coroner will choose to perform a post-mortem examination as part of their investigation if the cause of death is not clear. It is possible that in some cases where the clinical history is sufficiently clear (e.g., sudden onset severe headache in a patient with a known aneurysm) no post-mortem is carried out and the death certificate written based on the clinical history, but in the majority a post-mortem is undertaken.

For out-of-hospital events in the absence of PM or peri-mortem imaging it was assumed that patients with I60 code in the COD1 or COD_UNDERLYING position, or I61 in COD1 or COD_UNDERLYING plus I60 in COD2–7 had a rupture event. Other haemorrhage codes or diagnosis positions were not used to represent aneurysm rupture due to the inability to verify miscoded deaths assigned to other haemorrhage codes in the absence of imaging or post-mortem studies being performed.

### Analysis

Patients in the UHS cohort who subsequently sustained an aneurysm rupture were grouped as UHS EPR (rupture events identifiable from the UHS EPR) or non-EPR (rupture events identified only from HES-APC/CRD). The sensitivity and false negative rates using the combination of all codes (I60, I61, I62, S06, I46, R96 and R98) were calculated to determine their usefulness for screening. For additional PPVs of an ICD-10 code the first episode lasting greater than 1 day was chosen to represent a multi-episode continuous inpatient spell.

The definition of aneurysm rupture events matched the ISUIA Study [17]: definite (blood visualised on CT scan, post-mortem or intra-operative evidence of rupture), highly probable (symptoms of rupture plus CSF positive for xanthochromia) or probable (symptoms of rupture only). An out of hospital rupture death without a PM was classed as probable SAH. Equivocal cases underwent dual review between the lead (SH) and last author (DOB). For the purpose of analysis, all events (definite, highly probably, and probable) were classed as rupture events. The study aimed to determine the accuracy of national databases for identifying rupture of known UIA for the purpose of a natural history study, therefore any rupture events occurring in previously treated aneurysms or aneurysm rebleeds were excluded from analysis.

## Results

### University Hospital Southampton cohort

The UHS EPR search identified 1,544 patients with 2,031 aneurysms and is referred to as the UHS cohort. The most common symptoms/pathologies being investigated where the UIA was subsequently diagnosed were: subarachnoid haemorrhage from another source, including spontaneous and traumatic aetiologies, (n = 364), stroke (n = 267) and headache (n = 229). During the identification of the cohort from UHS EPR, 37 rupture events were noted in the EPR record after the recruitment date. The characteristics of the cohort are described in Table 1. The mean length of follow-up from aneurysm inclusion to cohort linkage was 6.27 ± 4.89 years.

**Table 1:** Characteristics of the University Hospital Southampton cohort. *Hypertension data complete for 1,407 patients with complete covariate coverage for the remainder of the table.

Patient characteristics	n = 1,544
Female	1,043 (67·6%)
Mean age (years)	58.7 (±12·6)
Hypertension	642 (45·6%)*
ADPKD	25 (1·6%)
Previous SAH	377 (24·4%)
Patients with multiple UIA	348 (22.5%)
Mean UIA per patient	1·32 ± 0·92
**Aneurysm characteristics**	**n = 2,031**
Aneurysm location •ACA •ICA •MCA •Vertebrobasilar	369 (18·2%) 699 (34·4%) 745 (36·7%) 218 (10·7%)
Mean aneurysm size (mm)	6·0 (±5·2)

### HES data - Confirmation of SAH events

**Fig 1:**
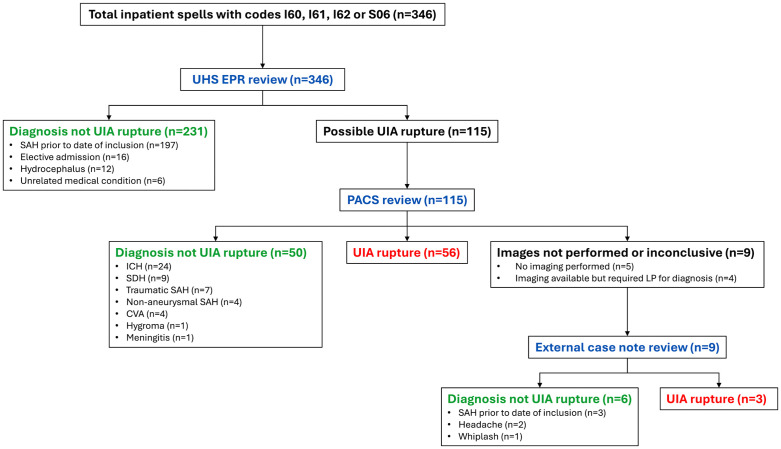
Final diagnoses of HES-APC inpatient spells identified by screening for cases of UIA rupture. The flowchart demonstrates the sequence where all identified cases underwent UHS EPR review first. Where these records showed that the event leading to admission was *not* rupture of the UIA under observation, no further review was performed. Images were requested for all cases in which UIA rupture remained possible to establish a final diagnosis. In nine cases either no imaging was performed (n = 5) or imaging did not resolve the diagnosis (n = 4) and case notes were obtained from the admitting hospital.

The HES database identified 1,193 episodes of which 771 episodes (346 continuous inpatient spells) were on/after the aneurysm inclusion date into the ROAR Study (Fig 1). No spells were identified using I46, R96 nor R98 codes. The rupture status for 231 continuous inpatient spells was confirmed from UHS EPR, 106 confirmed from imaging and 9 confirmed from external medical records. The final diagnoses reached at each step are shown in Fig 1. Of the 56 CT confirmed rupture events, six were ineligible for ROAR rupture analysis because the rupture occurred in a previously treated aneurysm (previously ruptured and treated aneurysms n = 3, ROAR aneurysms treated electively n = 3).

### HES-APC data – sensitivity of SAH codes

In view that in ROAR HES-APC will be used as a screening tool, and all potential rupture events identified from HES-APC will be individually verified, the sensitivity of HES-APC to identify rupture events and its false negative rates using the combination of all codes (I60, I61, I62, S06, I46, R96 and R98) were calculated.

Of the 37 rupture events noted in UHS EPR at the time of baseline data collection 35 were also found in HES-APC. Of the 37, 34 cases were managed in Southampton and 3 were admitted in other hospitals. Two rupture events were found in the UHS EPR but not HES-APC. One was correctly coded by the hospital as I62.9 (Intracranial haemorrhage (nontraumatic), unspecified) and the other incorrectly as I67.1 (Cerebral aneurysm, nonruptured). Both codes matched the diagnosis on the discharge summaries. It is not clear why the first patient was not identified by HES-APC given the correct coding; however, the second patient represents how an error in the original medical record propagates through to the rupture not being found in HES-APC. Of note, whilst the first of these patients was not found in HES-APC, they died in hospital and were correctly identified through CRD.

A further 18 rupture events in the UHS cohort were found in HES-APC which were not in the UHS EPR (Table 2). These patients with aneurysm rupture all were admitted to hospitals outside of Southampton. Without a reliable gold standard record of patients who were admitted to hospitals outside of Southampton rupture to compare against, it is not possible to report actual numbers of how many other rupture events occurred and that were missed from both HES-APC and UHS EPR. Therefore, we assumed coding practices and misclassification rates for HES-APC are similar across hospitals in England, and used the ratio of rupture events detected to those missed by HES-APC that were identified by UHS-EPR (2/37, 5.4%: 95% Confidence Interval -0.18 to 12.7) and applied this ratio to patients who presented to hospitals other than UHS. From this we estimate 1.03 rupture events would be missed by HES-APC and also not be found in UHS EPR during the study period (Table 2).

**Table 2:** Number of rupture events identified from UHS EPR or by HES-APC. ^*^This figure cannot be directly observed and has been estimated assuming the miscoding rate for patients admitted with aneurysm rupture to hospitals in England (not including Southampton) is the same as the observed misclassification rate for patients admitted with aneurysm rupture to Southampton (2/37).

	aSAH identified from UHS-EPR	aSAH not identified in UHS-EPR
aSAH identified in HES-APC	35	18
aSAH not identified in HES-APC	2	1^*^

### CRD data - In Hospital deaths

Three hundred and forty-eight deaths were identified by CRD in the UHS cohort. 77 of these contained a haemorrhage code (I60, I61, I62, S06) in any of the Cause Of Death data elements (COD1–7 or COD_UNDERLYING). No deaths were coded as I46, R96 or R98. Of these 77 deaths, 55 occurred in-hospital and underwent verification for the actual cause of death, of which 29 were confirmed as eligible and due to aneurysm rupture.

Of the 29 in-hospital deaths from aneurysm rupture, 25 (86.2%) had a corresponding record in HES-APC. Of the 4 in-patient deaths with no hospital admission, the medical records confirmed that two died in the emergency department without being admitted to hospital (this is consistent with HES-APC covering hospital admissions and not including emergency department activity). The third patient had cause of death established via PM and coroner inquest several weeks after their hospital discharge summary, and coding, was completed. The final rupture death was known in the EPR group with a diagnosis of I62.9 as discussed above. Twenty-six of these 29 in-hospital deaths were classed as *SAH – definite* based on imaging (n = 23) and post-mortem (n = 3), and 3 were *SAH – probable*.

There were no deaths known to EPR but missed in CRD and so it is assumed there are no deaths missing from the non-EPR column either (Table 3).

**Table 3:** Number of in-hospital deaths from aneurysm rupture identified by CRD, HES-APC and UHS EPR. * This figure cannot be directly observed and has been estimated assuming the miscoding rate for patients dying of aneurysm rupture in hospitals in England (not including Southampton) is the same as the observed misclassification rate for patients dying of aneurysm rupture in hospital Southampton (0/13).

	Rupture death known in UHS EPR (n = 13)	Rupture death not in UHS EPR (n = 16)
Rupture death in CRD + HES-APC	11	14
Rupture death in HES-APC only	0	0
Rupture death in CRD only	2	2
Rupture death in neither CRD nor HES-APC	0	0^*^

### CRD data - Out of hospital deaths

Twenty-two patients out of the 1,544 patients in the UHS cohort died out-of-hospital with an intra-cranial haemorrhage code in any COD position. Seventeen had I60 in either COD1 or COD_UNDERLYING of which 15 were confirmed to have been due to rupture of their UIA (*SAH-definite* on post-mortem n = 10, *SAH-probable* n = 5 where primary COD, i.e., COD1/COD_UNDERLYING, was I60). One patient’s SAH was ineligible due to their only ROAR eligible UIA being treated 12 years previously and the final patient had a non-aneurysmal ICH on a recent CT as an inpatient before dying at home.

Five patients had an intracranial haemorrhage code without I60 in either COD1 or COD_UNDERLYING. Of these, one with I61 in COD1 and COD_UNDERLYING with I60 in COD2 confirmed by PM and was considered as a rupture event. A second patient only had I61 codes in COD1 and COD_UNDERLYING, with no I60 code in any position and no PM. The next two had an I60 code in a COD position other than COD1 or COD_UNDERLYING and the primary COD was Urinary Tract Infection (N39.0) and Myocardial Infarction (I21.9) and the final patient had an S06 code in COD4 with X46 (accidental poisoning) in COD1.

**Fig 2:**
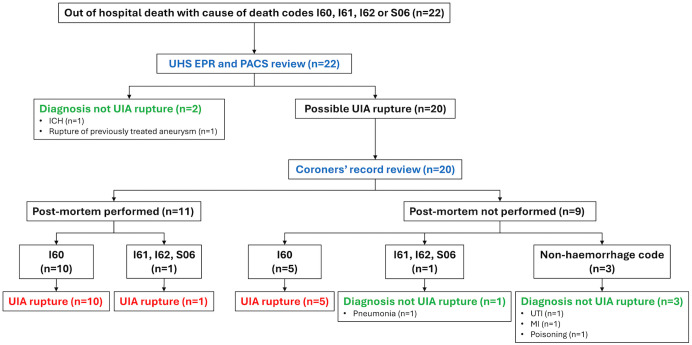
Final diagnoses of CRD out of hospital deaths identified by screening for cases of UIA rupture. The flowchart demonstrates the sequence where all identified cases containing the ICD-10 diagnosis code I60, I61, I62, or S06 anywhere in the death certificate underwent UHS EPR and available peri-death imaging review first. Where these records showed that the event leading to death was *not* rupture of the UIA under observation, no further review was performed. The county coroner records were reviewed for all cases in which UIA rupture remained possible to establish if the final diagnosis was made on post mortem examination. Cases whose cause of death (either COD1 or COD_UNDERLYING) were I60, or had post mortem confirmed evidence of aneurysm rupture, were deemed UIA rupture.

Overall, therefore, 16 patients had an out of hospital aneurysm rupture of which 11 (68·%) were confirmed by PM (Fig 2).

### Overall rupture detection rate

Of the 1,544 patients in the UHS cohort, HES-APC identified 53 rupture events subsequent to the UIA diagnosis. CRD identified a further 4 inpatient deaths and 16 out of hospital deaths from aneurysm rupture. The UHS EPR identified a further 1 aneurysm rupture which was not found in either HES-APC or CRD. The total number of confirmed rupture events in the UHS cohort was 74 out of 1,544 patients. Based on our assumption that diagnosis coding is similar across hospitals in England on average 1.03 patients would have suffered a rupture event but been missed from HES-APC and none would have been missed from in-patient CRD (Table 2 and Table 3).

The 16 rupture events resulting in out of hospital death, where the number of missed rupture events cannot be measured or estimated from a second source, were excluded from sensitivity calculation. The sensitivity of the national databases to detect aneurysm rupture resulting in admission to hospital was calculated as: (HES-APC identified rupture events + additional CRD identified rupture events)/ (HES-APC identified rupture events + additional CRD identified rupture events + additional UHS EPR identified rupture events + estimated rupture events missed by HES-APC, CRD and UHS EPR) = (53 + 4)/(53 + 4 + 1 + 1.03). Using this the sensitivity of the combined HES-APC and CRD search to detect aneurysm rupture events is 96.6% (CI: 91.2 to 100.0%). When combined with a local EPR search - (53 + 4 + 1)/ (53 + 4 + 1 + 1.03) - the sensitivity is 98.3% (CI: 95.0 to 100.0%).

### PPV and sensitivity of Individual ICD-10 codes

S1 File describes the sensitivity, specificity, PPV and NPV of each of the I60, I61, I62 and S06 codes for identifying aneurysm rupture events depending on their position in the list of diagnoses in HES-APC.

## Discussion

National digital databases are powerful research tools that collect vast amounts of data and are therefore ideal for large population studies. However, despite their demonstrated utility in neurosurgery [15], they remain subject to criticism from clinicians for the accuracy of their coding and consequently there is scepticism about their use to direct clinical decision making. We therefore set out to assess their sensitivity to detect events of aneurysm rupture in the context of the ROAR Study.

This study shows that combining codes and databases achieves very high sensitivities to detect intra-cranial aneurysm rupture events. This makes it ideal for the ROAR Study where the primary analysis is based on the EPR data collected at baseline combined with HES-APC/CRD which has a sensitivity of 98.3%. The ROAR cohort will be followed with 5 yearly updates using these national database searches and case review of potential rupture events which will still carry a 96.6% sensitivity. This represents a highly efficient and accurate way of following a cohort of this size over the ensuing decades. The 1.7% false negative rate (1-sensitivity) becomes clinically insignificant when considering that 5 year rupture rates are usually in single figures[2] a 2.00% rate becomes 2.03%.

It is important to note that using individual codes without verification would lead to an unacceptably low level of accuracy for this study. There are no previous estimates of the sensitivity of I60 codes to detect aneurysm rupture due to the difficulty in generating a gold standard to compare to. In this study the sensitivity of the I60 code as the primary diagnosis was only 84.7% for identifying rupture events which would therefore lead to a significant number of cases being missed. Six of the cases not identified by I60 were coded as ICH and one as SDH and none as sudden death or any other codes.

To identify all aneurysm rupture events, it is therefore necessary to search these additional codes to which patients with ruptured aneurysms may be correctly (in the case of pure aneurysmal ICH) or incorrectly coded (in cases of mixed ICH and SAH or pure SAH). The Million Women Study [13] found that 4.3% of patients with a HES diagnosis of I61 (intracerebral haemorrhage) actually had subarachnoid haemorrhage. This coding problem is considerably larger in a UIA cohort in whom a larger percentage of patients coded as ICH are likely to have had an aneurysmal rupture than a non-aneurysmal ICH. We have confirmed this is the case in our cohort where 43.2% of patients with ICH as the primary diagnosis code had an aneurysm rupture. Of these, 56.3% were accurately coded as ICH where the aneurysm rupture resulted in ICH with no concurrent SAH, and the remainder were miscoded where the primary diagnosis should have been I60 due to predominant SAH. The only way to detect these cases of aneurysm rupture without very high rates of false positives is to manually check the patient records flagged with ICH and SDH codes.

Similarly, the PPV of I60 (SAH) in the Million Women Study was 96% when compared to GP records and 96% when compared to hospital records, giving a false discovery rate of 4% (1-PPV). While this might be considered acceptable, the PPV in our population of patients with known UIA was much lower at 84%. This probably reflects that patients with UIA often have a previous history of SAH (from another aneurysm which leads to the diagnosis of an additional UIA) and thus the I60 code will appear more often as a co-morbidity than it would in the general population.

The high sensitivity therefore comes at the cost of a very high false discovery rate (16%), making validation of each case mandatory. However, in a disease like UIA with relatively low event rates, this is practical and feasible. Our methods to verify SAH events exactly mirror those in ISUIA and primary validation against CT scans/lumbar puncture results at time of SAH, or post-mortem. These would be expected to result in near 100% specificity. The advantage of our approach over studies like ISUIA is the efficiency with which these national databases can follow-up large cohorts, i.e., this pilot is already larger than ISUIA (74 rupture events in 10,153 patient/years vs 51 in 6,544 patient/years follow-up), and will allow the full cohort that is nearly 20 times larger to be followed up indefinitely.

### Strengths and limitations

The condition (aneurysm rupture) and the setting (United Kingdom) are ideally suited to the methodology described and address the major limitations that exist in any other study trying to address this clinical problem.

Aneurysm rupture is a binary event that would be expected to result in either hospital admission or death, with few exceptions. HES-APC and CRD provide universal coverage for these outcomes. Patients who die, are too frail or sick to be transferred to a neurosurgery unit, or have migrated around the country since the UIA diagnosis will therefore still be detected. It will also detect patients presenting late who will still more often than not eventually make contact with healthcare. Although, there are limitations to the coding, all patients and events are present in these databases. The Civil Registration Deaths database will allow identification of aSAH sudden deaths which occur in the pre-hospital setting and would otherwise be missed from hospital admission-based records. These solve the major problem in this field, in that patients who experience an aneurysm rupture do not necessarily present to the hospital managing their UIA. In fact, in this series half of rupture events were managed outside of the hospital managing the UIA (half managed in other hospitals and half suffering out of hospital death) and would be missed if local hospital records alone were used. This demonstrates the severe limitations to any series based only on clinically available follow-up.

One limitation is that although we are confident of coverage of rupture events that occurred in Southampton, we had to make estimates of the number of cases missed by HES-APC that were managed in other centres based on the assumption of similar coding practices between hospitals. ROAR will also use hospital admission data from Patient Episode Database for Wales (PEDW) and Scottish Morbidity Records (SMR), and death data from National Records for Scotland (NRS)-Deaths for rupture events occurring in Wales and Scotland. It is not known how the high sensitivity in the current data will generalise to patients identified in other home nations.

Manual case note review served as our gold standard in this study. Although we believe this was thorough, and additionally imaging was reviewed for all cases of rupture by a neurosurgeon with 10 years’ experience in neurosurgery, there remain limitations to this methodology. A prospectively followed cohort would be ideal for this purpose. A further limitation is that there is no practical gold standard to estimate rates of missed out of hospital deaths, or patients with rupture events who survived without attending hospital. This is inherent in any study of UIA in the absence of post-mortem examinations for all out of hospital deaths. However, out of hospital deaths attributable to aneurysm rupture have been defined and verified as in ISUIA where post-mortem was also not available in all patients. Importantly, CRD has not used any sudden death codes associated with an unexplained event (I46, R96 or R98) which would be the events which might have resulted in misclassification and overestimation of aneurysm rupture. Moreover, 69% of aneurysm rupture events leading to out of hospital death have been verified with a PM. It is also highly reassuring that the percentage of rupture events resulting in out of hospital death (post-mortem confirmed 11/74, 14.9% and total 16/74, 21.6%) are at least as frequent as was seen in the Finnish national FINRISK registry (18%) [18] and metanalysis of the literature (15–18%) [19]. Taken together, there is little to suggest any out of hospital deaths are being missed.

United Kingdom is ideally suited to this study as one of the largest countries to have a national database with complete coverage of hospital admissions. As an island nation with distinct borders there are no issues tracking patients who suffer aneurysm rupture at or near a border and are treated in a different country. There will be some cross border events due to foreign travel however, interestingly, HES-APC still captures many events occurring abroad when patients are repatriated for ongoing care. In this cohort, 3/74 rupture events (4.1%) occurred abroad which when compared to the Office of National Statistics figure of UK residents spending an average of 3.0% of the year abroad in 2022 [20] suggests we are capturing all, or nearly all, of the rupture events that occur abroad.

In addition to travel abroad, patients may emigrate during follow-up. HES records if a patient deregisters with their general practitioner. We anticipate that most, if not all, emigrating UIA patients deregister in order to have their notes and scans transferred. We therefore expect any missed events due to travel or migration to be at most low single digit percentages and which would only result in underestimates in aneurysm rupture rates measurable in hundredths of a percent.

## Conclusion

National databases for hospital admissions and deaths are a robust method for identifying aneurysm rupture, if used thoughtfully. The high sensitivity demonstrated here supports their use for outcome measurement in the Risk of Aneurysm Rupture Study and will enable natural history studies at least an order of magnitude larger than has been previously possible.

## Supporting information

S1 File Describes the sensitivity (Sen), specificity (Spec), positive predictive value (PPV) and negative predicative value (NPV) for each of the ICD-10 diagnosis codes for identifying episodes of aneurysm rupture based on the diagnosis code position within the HES record. For this purpose aneurysm rupture includes index aneurysm ruptures whereby patients with multiple aneurysms in whom the rupture of one aneurysm (index) led to the diagnosis of another unruptured aneurysm and inclusion of this UIA into the ROAR Study.
(DOCX)

## References

[1] Vlak MH, Algra A, Brandenburg R, Rinkel GJ. Prevalence of unruptured intracranial aneurysms, with emphasis on sex, age, comorbidity, country, and time period: A systematic review and meta-analysis. Lancet Neurol. 2011;10(7):626–36. doi: 10.1016/S1474-4422(11)70109-0 21641282

[2] Greving JP, Wermer MJH, Brown RD, Morita A, Juvela S, Yonekura M, et al. Development of the PHASES score for prediction of risk of rupture of intracranial aneurysms: A pooled analysis of six prospective cohort studies. Lancet Neurol. 2014;13(1):59–66. doi: 10.1016/S1474-4422(13)70263-1 24290159

[3] Juvela S, Poussa K, Lehto H, Porras M. Natural history of unruptured intracranial aneurysms: A long-term follow-up study. Stroke. 2013;44(9):2414–21. doi: 10.1161/STROKEAHA.113.001838 23868274

[4] Hall S, Birks J, Anderson I, Bacon A, Brennan PM, Bennett D, et al. Risk of Aneurysm Rupture (ROAR) study: Protocol for a long-term, longitudinal, UK multicentre study of unruptured intracranial aneurysms. BMJ Open. 2023;13(3):e070504. doi: 10.1136/bmjopen-2022-070504 36927598 PMC10030903

[5] Herbert A, Wijlaars L, Zylbersztejn A, Cromwell D, Hardelid P. Data resource profile: Hospital episode statistics admitted patient care (HES APC). Int J Epidemiol. 2017;46(4):1093. 28338941 10.1093/ije/dyx015 PMC5837677

[6] Tolonen H, Salomaa V, Torppa J, Sivenius J, Immonen-Räihä P, Lehtonen A, et al. The validation of the Finnish Hospital Discharge Register and Causes of Death Register data on stroke diagnoses. Eur J Cardiovasc Prev Rehabil. 2007;14(3):380–5. doi: 10.1097/01.hjr.0000239466.26132.f2 17568236

[7] McCormick N, Bhole V, Lacaille D, Avina-Zubieta JA. Validity of diagnostic codes for acute stroke in administrative databases: A systematic review. PLoS One. 2015;10(8):e0135834. doi: 10.1371/journal.pone.0135834 26292280 PMC4546158

[8] Hsieh MT, Huang KC, Hsieh CY, Tsai TT, Chen LC, Sung SF. Validation of ICD-10-CM diagnosis codes for identification of patients with acute hemorrhagic stroke in a national health insurance claims database. Clin Epidemiol. 2021;13:43–51. 33469381 10.2147/CLEP.S288518 PMC7813455

[9] Orso 9 M, Cozzolino F, Amici S, De Giorgi M, Franchini D, Eusebi P, et al. Validity of cerebrovascular ICD-9-CM codes in healthcare administrative databases. PLoS One. 2020;15(1):e0227653. 10.1371/journal.pone.0227653 PMC6952250 31918434

[10] Sedova P, Brown RD, Zvolsky M, Kadlecova P, Bryndziar T, Volny O, et al. Validation of Stroke Diagnosis in the National Registry of Hospitalized Patients in the Czech Republic. J Stroke Cerebrovasc Dis. 2015;24(9):2032–8. doi: 10.1016/j.jstrokecerebrovasdis.2015.04.019 26139454

[11] Woodfield R, Grant I, UK Biobank Stroke Outcomes Group, UK Biobank Follow-Up and Outcomes Working Group, Sudlow CLM. Accuracy of electronic health record data for identifying stroke cases in large-scale epidemiological studies: A systematic review from the UK Biobank Stroke Outcomes Group. PLoS One. 2015;10(10):e0140533. doi: 10.1371/journal.pone.0140533 26496350 PMC4619732

[12] Kirkman MA, Mahattanakul W, Gregson BA, Mendelow AD. The accuracy of hospital discharge coding for hemorrhagic stroke. Acta Neurol Belg. 2009;109(2):114–9. 19681442

[13] Wright FL, Green J, Canoy D, Cairns BJ, Balkwill A, Beral V, et al. Vascular disease in women: Comparison of diagnoses in hospital episode statistics and general practice records in England. BMC Med Res Methodol. 2012;12:161. doi: 10.1186/1471-2288-12-161 23110714 PMC3514155

[14] Wahba AJ, Cromwell DA, Hutchinson PJ, Mathew RK, Phillips N. Mortality as an indicator of quality of neurosurgical care in England: A retrospective cohort study. BMJ Open. 2022;12(11):e067409. doi: 10.1136/bmjopen-2022-067409 36332948 PMC9639111

[15] Wahba AJ, Cromwell DA, Hutchinson PJ, Mathew RK, Phillips N. Assessing national patterns and outcomes of pituitary surgery: Is hospital administrative data good enough?. Br J Neurosurg. 2023;37(5):1135–42. doi: 10.1080/02688697.2023.2170982 36727284

[16] England N. Technical details of the data linkage algorithm, 2024. https://digital.nhs.uk/services/personal-demographics-service/master-person-service/the-person_id-handbook/technical-details-of-the-data-linkage-algorithm. 2024.

[17] Wiebers DO, Whisnant JP, Huston J 3rd, Meissner I, Brown RD, Piepgras DG, et al. Unruptured intracranial aneurysms: Natural history, clinical outcome, and risks of surgical and endovascular treatment. Lancet. 2003;362(9378):103–10. doi: 10.1016/s0140-6736(03)13860-3 12867109

[18] Lindbohm JV, Kaprio J, Jousilahti P, Salomaa V, Korja M. Risk factors of sudden death from subarachnoid hemorrhage. Stroke. 2017;48(9):2399–404. 28739833 10.1161/STROKEAHA.117.018118

[19] Huang J, van Gelder JM. The probability of sudden death from rupture of intracranial aneurysms: A meta-analysis. Neurosurgery. 2002;51(5):1101–5. doi: 10.1097/00006123-200211000-00001 12383354

[20] O.o.N. Statistics, Travel Trends 2022, in: O.o.N. Statistics (Ed.) Office of National Statistics, 2023.

