# Supplementary material for: Use of UK national health databases for detecting intra-cranial aneurysm rupture in the Risk of Aneurysm Rupture (ROAR) study
Source: PLOS Digit Health. 2026 May 18;5(5):e0001388. doi: 10.1371/journal.pdig.0001388 (PMC13183183; doi:10.1371/journal.pdig.0001388)
Supplement: S1 File — For this purpose aneurysm rupture includes index aneurysm ruptures whereby patients with multiple aneurysms in whom the rupture of one aneurysm (index) led to the diagnosis of another unruptured aneurysm and inclusion of this UIA into the ROAR Study. (DOCX) [file pdig.0001388.s001.docx]

**S1 File**

S1 File describes the sensitivity (Sen), specificity (Spec), positive predictive value (PPV) and negative predicative value (NPV) for each of the ICD-10 diagnosis codes for identifying episodes of aneurysm rupture based on the diagnosis code position within the HES record. For this purpose aneurysm rupture includes index aneurysm ruptures whereby patients with multiple aneurysms in whom the rupture of one aneurysm (index) led to the diagnosis of another unruptured aneurysm and inclusion of this UIA into the ROAR Study.

| ICD-10 | Diagnosis position 1 | | Diagnosis position 1-2 | | Diagnosis position 1-3 | | | Diagnosis position 1-20 | |
| --- | --- | --- | --- | --- | --- | --- | --- | --- | --- |
| I60 | TP = 229 | Sen = 87.7 | TP = 239 | Sen = 91.6 | | TP = 241 | Sen = 92.3 | TP = 242 | Sen = 92.7 |
|  | FP = 29 | Spec = 66.6 | FP = 39 | Spec = 55.2 | | FP = 41 | Spec = 52.9 | FP = 46 | Spec = 47.1 |
|  | TN = 58 | PPV = 88.8 | TN = 48 | PPV = 86.0 | | TN = 46 | PPV = 85.5 | TN = 41 | PPV = 84.0 |
|  | FN = 32 | NPV = 64.4 | FN = 22 | NPV =68.6 | | FN = 20 | NPV =69.7 | FN = 19 | NPV =68.3 |
| I61 | TP = 18 | Sen = 6.9 | TP = 39 | Sen = 14.9 | | TP = 46 | Sen = 17.6 | TP = 51 | Sen = 19.5 |
|  | FP = 20 | Spec = 77.0 | FP = 25 | Spec = 71.3 | | FP = 26 | Spec = 70.1 | FP = 27 | Spec = 69.0 |
|  | TN = 67 | PPV = 47.4 | TN = 62 | PPV = 60.9 | | TN = 61 | PPV = 63.9 | TN = 60 | PPV = 65.4 |
|  | FN = 243 | NPV = 21.6 | FN = 222 | NPV = 22.0 | | FN = 215 | NPV = 22.1 | FN = 210 | NPV = 22.2 |
| I62 | TP = 4 | Sen = 1.5 | TP = 5 | Sen = 1.9 | | TP = 6 | Sen = 2.3 | TP = 8 | Sen = 3.1 |
|  | FP = 5 | Spec = 94.3 | FP = 9 | Spec = 89.7 | | FP = 10 | Spec = 88.5 | FP = 12 | Spec = 86.2 |
|  | TN = 82 | PPV = 44.4 | TN = 78 | PPV = 35.7 | | TN = 77 | PPV = 37.5 | TN = 75 | PPV = 40.0 |
|  | FN = 257 | NPV = 24.2 | FN = 256 | NPV = 23.4 | | FN = 255 | NPV = 23.2 | FN = 253 | NPV = 22.9 |
| S06 | TP = 0 | Sen = 0.0 | TP = 0 | Sen = 0.0 | | TP = 0 | Sen = 0.0 | TP = 0 | Sen = 0.0 |
|  | FP = 9 | Spec = 89.7 | FP = 9 | Spec = 89.7 | | FP = 9 | Spec = 89.7 | FP = 10 | Spec =88.5 |
|  | TN = 78 | PPV = 0.0 | TN = 78 | PPV = 0.0 | | TN = 78 | PPV = 0.0 | TN = 77 | PPV = 0.0 |
|  | FN = 261 | NPV = 23.0 | FN = 261 | NPV = 23.0 | | FN = 261 | NPV = 23.0 | FN = 261 | NPV = 22.8 |
